# Supplementary material for: Comparative Analysis of G-Layers in Bast Fiber and Xylem Cell Walls in Flax Using Raman Spectroscopy
Source: Biomolecules. 2023 Feb 24;13(3):435. doi: 10.3390/biom13030435 (PMC10046372; doi:10.3390/biom13030435)
Supplement: Supplementary file 1 [file biomolecules-13-00435-s001.zip › Table S1 Proof.pdf]

Table S1: Peak assignment to chemical bonds and relative intensities in Raman average spectra. Assignment of peaks was done for xylem G-layer comparatively with control BF or control wood average spectra. Intensity of the peak is evaluated as follows: vw= very weak, w: weak, m: medium, s: strong, vs: very strong, trace: usually a shoulder or broad band with small intensity. Presence +, Absence ø. XG: xyloglucan. GlcM: glucomannan. Dark grey colored-boxes highlight specificity.

Peak (cm^{-1})	Peak in literature (cm-1)	assignment	Putative polymers	G-layer Tension side	G-layer Opposite side	Bast Fibers	Control Wood	References
**380**	377–379–380–382	δ(CCC), symmetric ring deformation	Cellulose	+(s)	+(s)	+(s)	+(w)	[22]-[25]
**405**			Cellulose	+(vw)	+(vw)	+(vw)	+(vw)	[22]
**435**	434–435–437	δ(COC), δ(CCC), ring deformation	Cellulose	+(m)	+(m)	+(m)	+(vw)	[22]-[24]
**462**		δ(COC), δ(CCC), ring deformation, xylan	Xylan	+(m)	+(m)	+(m)	+(vw)	[24]
**490**	489–490–492–496	δ(COC), glycosidic linkage, xylan	Xylan	+(m)	+(m)	+(m)	+(vw)	[23]-[24]
**517**	517–518–519–521	δ(COC), glycosidic linkage/CCC ring deformation	Xylan, XG	+(s)	+(s)	+(s)	+(vw)	[22]-[25]
**562**	565–567–575	δ(COC), ring pyranose	Cellulose Xylan	+(w)	+(w)	+(w)	+(vw)	[24]
**607**	602–607–609–611	δ(CCH)	Cellulose	+(vw)	+(vw)	ø	ø	[24]
**650**		δO-H out of plane bending mode	Crystalline cellulose	+(vw)	+(vw)	ø	ø	[22]-[24]
**900**	893–900–914	δ(HCC), δ(HCO) cluster of peaks-methine bending	Cellulose	+ (m)	+ (m)	+(m)	+(vw)	[22]-[24]
**969**	966–968–971–974	ρ(CH_2) skeletal	β-glucan	+(m)	+(m)	+(m)	+(vw)	[22]-[24]
**990**	993–995–997–999	ρ(CH_2)	Arabinose	+(w)	+(w)	+ (vw)	+(vw)	[22]-[24]
**1,094**	1,091-1,092-1,095-1,096	xyloglucan β (1-4) linked glucose	Cellulose xylan, XG, GlcM	+(s)	+(s)	+(s)	+(m)	[17], [22]-[26]
**1,126**	1,118–1,121	ν(COC) symmetric, glycosidic ring breathing	Xylan Cellulose	+(s)	+(s)	+(s)	+(vw)	[17],[23],[27]
**1,150**	1,147–1,150–1,152–1,154	ν(CC), ν(CO) asymmetric, ring breathing (glucopyranose)	Cellulose	+(s)	+(s)	+(s)	+(m)	[17],[23],[24]
**1,203**	1,200-1,202	Lignin methoxy vibrations	Lignin	+(vw)	+(vw)	+(vw)	+(vw)	[28]

**1,270**	1,272	Aryl-O stretching of aryl-OH and aryl-O-CH_3 (G unit)	a1: G-unit lignin	+(m)	+(m)	ø	+(m)	[22],[28]
**1,295**	1,292–1,293–1,294	δ(CH_2) twisting long chain	Aromatics, Lignin	+(w)	+(w)	trace	+(m)	[22],[24],[29]
**1,334**	1,331-1,332	OH in plane bending	Cellulose	+ (m)	+ (m)	+(m)	+(m)	[17],
**1,376**	1,378–1,379–1,380	δ(CH2)	Cellulose	+(s)	+(s)	+(s)	+(s)	
**1,421**		Lignin methoxy deformation	a2: aromatics	+(vw)	+(vw)	ø	+(m)	[22]-[25]
**1,452**	1,453–1,455	δ(CH2) symmetric bending on pyranose ring	Hemicelluloses Amorphous cellulose, pectin	+(w)	+(w)	+(w)	+(m)	[22],[26],[27]
**1,480**	1,478	δ(CH2) scissors	Cellulose	+(vw)	+(vw)	+(vw)	+(m)	[22],[24]
**1,599**	1,593–1,601	ν(C=C), aromatics	a3: coniferyl aldehyde Lignin	++(s)	++(s)	trace	+++(vs)	[23],[27]
**1,658**	1,657–1,660	δ(CC), C=O coniferyl aldehyde, C=C coniferyl alcohol	a4: coniferyl alcohol, conferyl aldehyde Lignin	++(s)	++(s)	trace	+++(vs)	[17]
**1,729**	1,725-1,732	C=O of acetyl or carboxylic acid group	Hemicelluloses	+(w)	+(w)	trace	+(w)	[17],[27]
