# Supplementary figures and images for: Comparative Analysis of G-Layers in Bast Fiber and Xylem Cell Walls in Flax Using Raman Spectroscopy
Source: Biomolecules. 2023 Feb 24;13(3):435. doi: 10.3390/biom13030435 (PMC10046372; doi:10.3390/biom13030435)

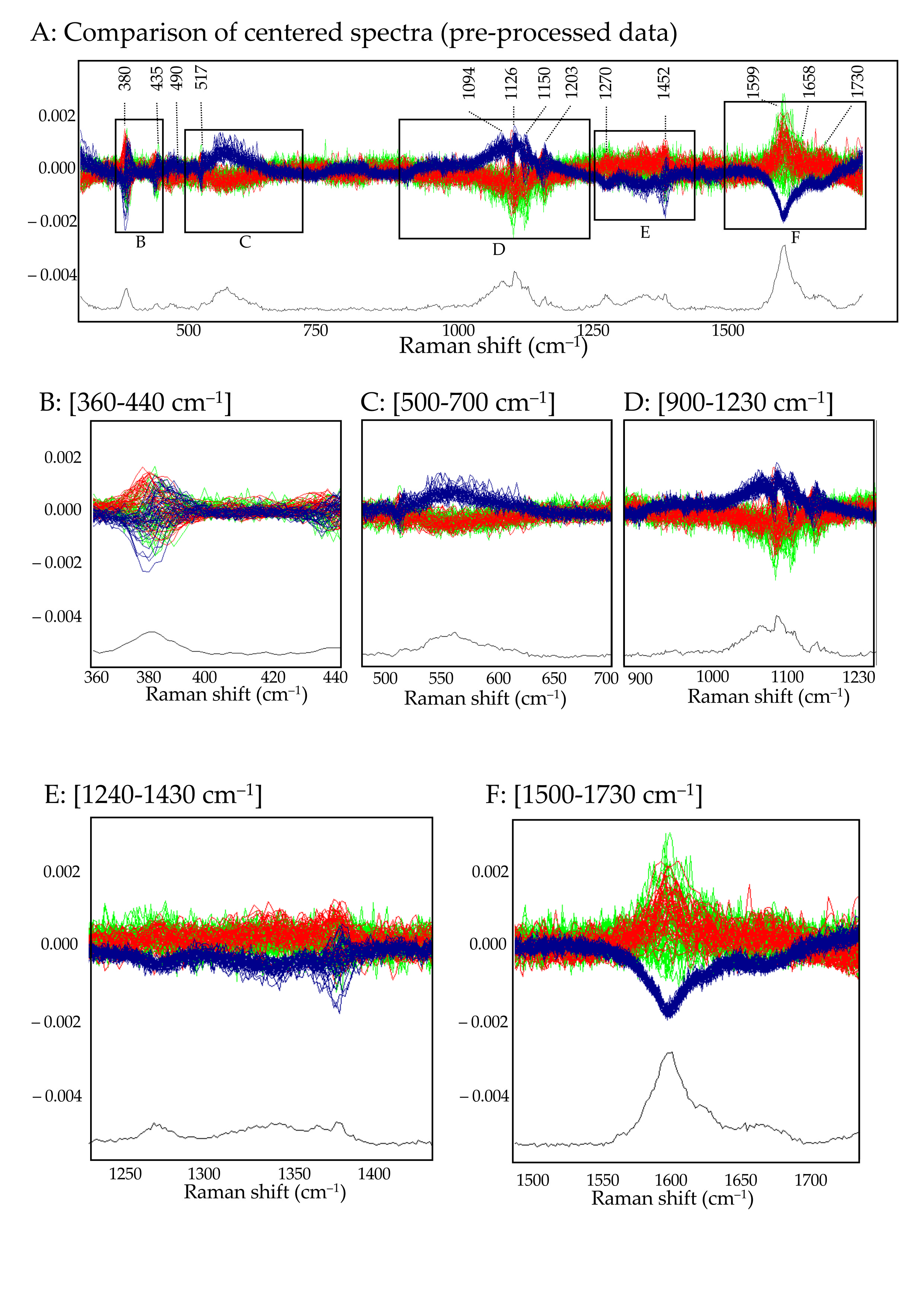

Supplement: Supplementary file 1 [file biomolecules-13-00435-s001.zip › Figure S1.tif]
